# Patients’ Expectations of Doctors’ Clinical Competencies in the Digital Health Care Era: Qualitative Semistructured Interview Study Among Patients

**DOI:** 10.2196/51972

**Published:** 2024-08-27

**Authors:** Humairah Zainal, Xin Xiao Hui, Julian Thumboo, Warren Fong, Fong Kok Yong

**Affiliations:** 1 Health Services Research Unit Singapore General Hospital Singapore Singapore; 2 Department of Rheumatology and Immunology Singapore General Hospital Singapore Singapore; 3 Duke-NUS Medical School National University of Singapore Singapore Singapore; 4 Department of Medicine Yong Loo Lin School of Medicine National University of Singapore Singapore Singapore

**Keywords:** digital health, clinical competence, patient engagement, qualitative research, Singapore, mobile phone

## Abstract

**Background:**

Digital technologies have impacted health care delivery globally, and are increasingly being deployed in clinical practice. However, there is limited research on patients’ expectations of doctors’ clinical competencies when using digital health care technologies (DHTs) in medical care. Understanding these expectations can reveal competency gaps, enhance patient confidence, and contribute to digital innovation initiatives.

**Objective:**

This study explores patients’ perceptions of doctors’ use of DHTs in clinical care. Using Singapore as a case study, it examines patients’ expectations regarding doctors’ communication, diagnosis, and treatment skills when using telemedicine, health apps, wearable devices, electronic health records, and artificial intelligence.

**Methods:**

Findings were drawn from individual semistructured interviews with patients from outpatient clinics. Participants were recruited using purposive sampling. Data were analyzed qualitatively using thematic analysis.

**Results:**

Twenty-five participants from different backgrounds and with various chronic conditions participated in the study. They expected doctors to be adept in handling medical data from apps and wearable devices. For telemedicine, participants expected a level of assessment of their medical conditions akin to in-person consultations. In addition, they valued doctors recognizing when a physical examination was necessary. Interestingly, eye contact was appreciated but deemed nonessential by participants across all age bands when electronic health records were used, as they valued the doctor’s efficiency more than eye contact. Nonetheless, participants emphasized the need for empathy throughout the clinical encounter regardless of DHT use. Furthermore, younger participants had a greater expectation for DHT use among doctors compared to older ones, who preferred DHTs as a complement rather than a replacement for clinical skills. The former expected doctors to be knowledgeable about the algorithms, principles, and purposes of DHTs such as artificial intelligence technologies to better assist them in diagnosis and treatment.

**Conclusions:**

By identifying patients’ expectations of doctors amid increasing health care digitalization, this study highlights that while basic clinical skills remain crucial in the digital age, the role of clinicians needs to evolve with the introduction of DHTs. It has also provided insights into how DHTs can be integrated effectively into clinical settings, aligning with patients’ expectations and preferences. Overall, the findings offer a framework for high-income countries to harness DHTs in enhancing health care delivery in the digital era.

## Introduction

### Background

Digital health care, which can be defined as the use of advanced technologies to replace or complement existing health care services and practices, is becoming increasingly prevalent in clinical work [1]. Digital health care technologies (DHTs) such as electronic health records (EHRs), telemedicine, wearable devices, mobile health, and other digital tools have been beneficial to health care delivery [2]. The widespread adoption of EHRs has enabled the streamlining of patient data, making it easily accessible to health care providers and improving care coordination [3]. The growth of telemedicine has facilitated consultations between health care providers and patients living in remote and underserved areas [4,5]. Furthermore, the development of wearable sensors and smartphone apps has allowed for the continuous monitoring of neurodegenerative disorders and the detection of related disease symptoms, respectively, among other benefits [6]. The integration of automation and artificial intelligence (AI) in health care has also enhanced various medical procedures including improving diagnosis and overall work efficiency [1,7]. These technologies have proven useful for patients especially where the barriers to adoption are low [1,7].

Singapore, a high-income country in Southeast Asia, makes a compelling case study for investigating how end users perceive the use of DHTs. This is attributed to a myriad of reasons: its high rate of technological advancement, strong government support for digitalization, efficient health care system, and a diverse population. Singapore is renowned for its advanced technological infrastructure and digital capabilities. Despite being the smallest country in Southeast Asia [8], Singapore is ranked first in the world in internet availability [9]. In addition, there is a high proliferation of digital technology and mobile phones in the country [10]. In 2020, 88% of its population was using smartphones compared to 78% globally [9]. A survey has also shown that the potential for digital uptake is high, and Singaporeans are open to incorporating digital tools in their health routines especially if it is beneficial to them [11]. The Singapore government has also invested heavily into transforming Singapore into a Smart Nation, an initiative that leverages technology and data to improve economic competitiveness and enhance the lives of its citizens [12]. In the realm of health care, it has rolled out digital initiatives to fulfill its population’s health care needs [9]. These include introducing telehealth, as well as implementing robotics and assistive technology solutions, to help older adults and those with mobility issues [13]. AI technologies have also been used in specialties such as radiology and ophthalmology and are expected to be a transformational force in Singapore’s health care [14]. Its well-established health care system makes it an ideal environment to study the integration of DHTs into health care delivery. Notwithstanding its small size of 734 km^2^, Singapore is also home to a diverse population with varying demographics, including different cultural influences, age groups, and socioeconomic backgrounds [8]. These factors make Singapore a worthwhile case study to explore patient perceptions and expectations of their doctors’ DHT competencies.

Despite the proliferation of DHTs in many high-income countries such as Singapore, there is a lack of research that captures the perspectives of patients on the clinical competencies that doctors need to be equipped with when DHTs are being deployed. Clinical competencies in this study refer to the knowledge, skills, and attitudes that medical doctors should possess when assessing, diagnosing, treating, and caring for their patients [15,16]. Existing research on patient and public involvement in digital health has either taken a broad approach [17,18] or is focused on specific areas, such as digital health consent in the context of clinical care [19]. It is important to examine patients’ expectations of doctors’ competencies in DHTs in their clinical care for several reasons. First, it would help identify any existing gaps in competencies. Second, patients’ confidence in the health care system would depend in part on doctors’ using DHTs competently in the diagnosis and management of their medical conditions. Third, studies and reviews have shown that greater efforts to involve patients in digital planning and implementation need to be made from the outset so that they can contribute meaningfully to digital innovation initiatives [17,18].

### Objective

This paper aims to address the aforementioned gap by assessing how patients perceive the use of DHTs by doctors in the context of clinical care. Using Singapore as a case study, it examines patients’ expectations of their doctors when telemedicine, health apps, wearable devices, EHRs, and AI technologies are used. Specifically, it evaluates their views on how doctors should communicate, diagnose, and treat their conditions when deploying these DHTs. In view of the lack of research done in non-Western contexts on patient involvement in digital health studies [17,19], Singapore represents an important case study for exploring this topic.

## Methods

### Setting and Sample

We adopted a social constructivist approach to understanding the subjective experiences and diverse interpretations of individuals in their social context. This approach facilitates the understanding of the meaning of a text as an “interaction between the preconceptions of the reader and the intentions of the producer” [20]. Hence, it raises the possibility of an agreement between various individual meaning constructions [20]. A qualitative study involving individual semistructured interviews with patients from outpatient clinics of a public health care cluster in Singapore was performed.

Data were collected from June 2022 to June 2023 through individual interviews with patients. For maximum variation, purposive sampling was used to recruit patients who were seeking treatment for various medical conditions. This type of sampling has been widely adopted in many health sciences research studies, as it enables researchers to select participants with specific characteristics or conditions relevant to the study [21-23]. Accordingly, 1 to 2 patients with at least 1 of the conditions that constituted chronic disease burden in Singapore were recruited [24]. These were the medical conditions that were commonly reported in Singapore and that would have a significant impact on health [24]. They included cancer, cardiovascular disease, diabetes and kidney diseases, neurological diseases, and autoimmune diseases. Amid the trend of an aging population with comorbidities in high-income countries [25], it is imperative to examine the type of clinical competence that may fulfill the health care needs of patients with multiple conditions. In addition, participants from different age groups, spanning between 20 and 39 years, 40 and 59 years, and ≥60 years, were interviewed. This was done with the aim of exploring whether patients from different generational cohorts had varying expectations regarding their doctors’ competencies and if so, what those expectations were. Furthermore, we recruited patients from various ethnic groups to assess if cultural factors had any influence on what patients anticipate of their doctors.

We first conducted pilot interviews with 5 patients from the outpatient clinics of a health care cluster who met the aforementioned criteria to ensure that the interview questions were clear and relevant and that the interview process flowed smoothly. We then carried out more interviews until data saturation was reached. Potential participants were first identified by WF before research fellow HZ sent email invitations for the interview. Interviews were then conducted and recorded over Zoom (Zoom Video Communications) unless participants requested an in-person interview. Acceptance of invitations for a Zoom interview served as consent for participation. In the reporting of findings, we followed the Standards for Reporting Qualitative Research proposed by O’Brien et al [26] (Table 1).

**Table 1:** Standards for reporting qualitative research.

Number			Topic		Item
**Title and abstract**					
	S1	Title		“Patients’ Expectations of Doctors’ Clinical Competencies in the Digital Health Care Era: Qualitative Semistructured Interview Study Among Patients”	
	S2	Abstract		Please refer to the Abstract in the main text.	
**Introduction**					
	S3	Problem formulation		The study aims to explore patients’ expectations of doctors’ clinical competencies when using DHTs^a^ in medical care, using Singapore as a case study.	
	S4	Research questions		What are some of the patients’ expectations of and concerns with doctors’ clinical competencies when DHTs such as telemedicine, health apps, wearable devices, EHRs^b^, and AI^c^ are being deployed? What are their views on how doctors should communicate, diagnose, and treat their conditions when deploying these DHTs in clinical care?	
**Methods**					
	S5	Qualitative approach and research paradigm		This manuscript is based on an ethnographic study involving individual semistructured interviews with patients from the outpatient clinics of a health care cluster in Singapore. It adopts a social constructivist approach and analyzes the data inductively using the 6-step process given by Braun and Clarke [27].	
	S6	Researcher characteristics and reflexivity		Please refer to the Data Collection section for elaboration.	
	S7	Context		The interviews were conducted after the COVID-19 cases showed a declining trend in Singapore. At the peak of the COVID-19 pandemic, health care settings witnessed an acceleration in the adoption of digital technologies in health care, such as the use of AI to detect the severity of pneumonia in COVID-19 patients, telemedicine for remote consultation, and robots to deliver medication in hospital wards.	
	S8	Sampling strategy		After identifying the illnesses that constituted chronic disease burden in Singapore, we conducted pilot interviews with 5 patients from the outpatient clinic of a health care cluster before determining the required sample size. The latter was done by identifying 1 to 2 patients who were having at least 1 of the illnesses from each of the following age bands: 20-39 years, 40-59 years, and ≥60 years.	
	S9	Ethical issues pertaining to human participants		Waiver for ethical approval was granted by SingHealth Centralised Institutional Review Board (reference 2020/2880).	
	S10	Data collection methods		Please refer to the Methods section in the main text for details.	
	S11	Data collection instruments and technologies		22 interviews were conducted and recorded over Zoom, while 3 were done in person (upon participants’ request). The latter interviews were audio recorded. Each interview lasted approximately 40 minutes.	
	S12	Units of study		25 patients who were experiencing any one of the illnesses that constitute chronic disease burden in Singapore took part in the once-off interview.	
	S13	Data processing		The interviews were transcribed verbatim by a transcriber, and the transcripts reviewed by HZ and FKY to ensure transcription accuracy. To protect participants’ anonymity, we assigned code identifiers beginning with “P” to each of them, an abbreviation for “patients.”	
	S14	Data analysis		The researchers adopted an inductive thematic analysis approach when evaluating the data to draw common and shared meanings among participants. Coding frameworks and themes were developed iteratively using the 6-step process proposed by Braun and Clarke [27].	
	S15	Techniques to enhance trustworthiness		Collecting data from patients with different medical conditions Comparing the findings with those of studies conducted in other high-income countries that are facing similar pace of digital transformations in health care Comparing the findings with up-to-date published data on patients’ expectations of doctors and on the views of stakeholders from the health care industry toward the digital competencies needed for current and future clinical practice.	
**Results and findings**					
	S16	Synthesis and interpretation		Refer to the Principal Findings section.	
	S17	Links to empirical data		Refer to illustrative quotes in the Results section. Full data are available upon reasonable request to authors.	
**Discussion**					
	S18	Integration with prior work, implications, transferability, and contributions to the field		Refer to the Discussion section.	
	S19	Limitations		Refer to the text under Strengths and Limitations.	
**Other**s					
	S20	Conflicts of interest		None was reported by the authors.	
	S21	Funding		SingHealth Duke-NUS Medicine Academic Clinical Programme under Seah Cheng Siang Distinguished Professorship in Medicine.	

^a^DHT: digital health care technology.

^b^EHR: electronic health record.

^c^AI: artificial intelligence.

### Data Collection

The interview guide was developed based on the framework given by Kallio et al [28] for developing a qualitative semistructured interview guide. This involved a 5-step process, namely, identifying the prerequisites to use a semistructured interview, retrieving and using previous knowledge in the literature and empirical data based on consultations with experts, formulating the preliminary interview guide with the research team, pilot-testing the interview with 5 patients, and finalizing the full interview guide for data collection [28]. Data from patients with different medical conditions and social backgrounds were then collected to ensure a broad representation of findings. At the beginning of the discussion, the interviewer reiterated the participants’ right to withdraw from the study at any time during the interview. In addition, the interviewer informed the participant that the data collected up to the point of withdrawal would be retained and analyzed to enable a comprehensive evaluation of the findings.

The interview questions sought patients’ views on their experiences with DHTs, if any; their expectations of their doctors when it comes to treating and diagnosing their illnesses, particularly when DHTs were used; and their suggestions on how different stakeholders in the health care system could improve the way patients were treated and diagnosed with the aid of DHTs (Table 2). The DHTs that we selected were those that were reported to be popular among Singaporeans and that were expected to transform the health care delivery of high-income countries such as Singapore [11,29]. These included health care–related mobile apps. In Singapore, the app that is commonly used by Singaporeans is the HealthHub app. Launched in 2015, it is a one-stop online health information and services portal and mobile app that allows Singaporeans to access their health records, laboratory results, and other health-related information; manage future medical appointments; and pay medical bills, among other functions [9]. The other DHTs were wearable devices, EHRs, AI, and telemedicine, which we defined as the use of information and communications technologies to deliver health care services from a distance [30].

In reviewing the issues of reflexivity, we considered how our assumptions and prejudices might influence our research [31]. In so doing, we discovered that there was a tendency to assume that participants of higher educational qualifications would be more familiar with the terms related to DHTs. Hence, the assumption is that there would be less need for the interviewer, HZ, to explain the terms in detail. However, the pilot interviews proved otherwise. To overcome this bias, HZ defined the key terms found in the interview guide for all participants for subsequent interviews.

**Table 2:** Interview questions.

Number	Questions
1	We are living in an age where digital technologies are prevalent in our everyday lives including in our health care experience. New forms of technology such as Artificial Intelligence or AI and robotics have influenced health care treatment and diagnosis. Do you expect your doctor’s roles to change with the advent of digital technologies? If yes, in what ways? What are your expectations of a doctor when it comes to (1) treating your illness or conditions, (2) diagnosis, and (3) overall patient care?
2	Do you have any experience of using digital platforms when consulting your doctor? These include video consultations over Zoom, WhatsApp or any other teleconferencing platforms. If yes, please share with me your experience, particularly with regard to the quality of care you received. What role do you expect your doctor to play during a teleconsultation? Do you prefer face-to-face or virtual consultation with your doctor? Why? There are certain limitations of using virtual consultation, such as the absence of physical examination. Do limitations like this matter to you?
3	Do you have any experience of using other digital tools for health care, particularly a medical monitoring device or wearable device? If yes, share with me your experience. Did you face any inconveniences or challenges when using these devices? How did you overcome them? What role do you expect your doctor to play when you are using these devices?
4	Have you used any health care-related smartphone applications such as “HealthHub,” to seek health care services? (HealthHub is a one-stop web portal with an accompanying digital application for obtaining information about health conditions, assessing health records, managing medical appointments, and paying medical bills, among other functions). If yes, share with me your experience. Did you face any inconveniences when using the app? If no, are you open to using such apps for health care purposes?
5	In your opinion, how important is it for doctors to have the ability to interact with their patients and caregivers/family members, including explaining medical terms and giving medical advice clearly? How important is it for doctors to have good inter-personal skills such as showing respect, care and compassion for their patients? How important is it for doctors to maintain eye contact with you while he/she is keying in your medical records in the system? Why?
6	I would like to know your thoughts on what the following stakeholders can do in order to improve patients’ experience when digital technologies are being used: Doctors Hospitals and polyclinics (public primary health care clinics) Medical schools (particularly in terms of training future medical graduates in digital technologies)
7	Do you have any other concerns if your doctor were to use digital technologies to treat you or to diagnose your medical condition?
8	Do you have any other comments on the skills or knowledge that doctors need to have when treating, diagnosing, assessing, and caring for you especially amid rapid technological advances?

### Data Analysis

HZ and FKY read the transcripts independently and adopted an inductive thematic analysis approach when evaluating the data. This allowed us to draw common and shared meanings among the participants [32]. It also enabled the flexibility to accommodate new insights about the data [33]. Coding frameworks and themes were developed iteratively using the 6-step process proposed by Braun and Clarke [27] in which we first familiarized ourselves with the data by reading the transcripts in their entirety before generating relevant codes and combining them into themes. We then reviewed the themes before determining those that we deemed significant to the research questions and finally reporting the findings [27].

We also compared the findings with all relevant up-to-date local and global literature that report on patients’ expectations of doctors, as well as views of stakeholders from the health care industry toward the competencies needed for current and future clinical practice [34-39]. Any coding discrepancies were resolved through consensus between HZ and FKY and through seeking the opinions of other coauthors.

### Ethics Approval

Waiver for ethical approval was granted by SingHealth Centralised Institutional Review Board (reference 2020/2880).

## Results

A total of 25 patients of different genders; from different ethnicities, socioeconomic backgrounds, educational levels, age bands; and with various medical conditions participated in our study. Their demographics are described in Table 3. In summary, 14 (56%) were male and 11 (44%) were female. Only 8 (32%) out of 25 patients had used telemedicine, while 17 (68%) patients had used a medical monitoring or wearable device. As many as 18 (72%) participants had used the HealthHub app. Generally, the participants were receptive to the use of DHTs as long as their safety and personal medical data were not compromised.

With regard to wearable devices and mobile apps such as HealthHub, most participants (n=18, 72%) regardless of gender, ethnicity, age, socioeconomic background, and educational qualifications expected their doctors to be competent and more proactive in engaging them with the medical data found in these devices. They also expected doctors to handle the data in an ethical manner, in a way that abided by personal data protection laws. Specifically, participants such as P20 would like their doctors to offer them guidance on using medical devices correctly, review the results of laboratory tests with them, and explain any anomalies that may be present in their medical data (Table 4). There were also participants such as P5 who expected doctors to collaborate with patients in their health care journey through advising them on the type of apps that were safe to use instead of taking on paternalistic doctor-patient roles (Table 4). Others, such as P24, expected doctors to comply with the guidelines on data protection. Amid increased cybersecurity risks, she was concerned that her personal information might get leaked when doctors use mobile apps such as WhatsApp to share photos of patients’ medical scans and other medical information with their colleagues (Table 4).

Regarding telemedicine, participants expected a level of assessment of their medical conditions that was similar to a physical consultation. Specifically, participants such as P22 expected their doctors to deliver comprehensive quality of care that would not compromise their safety when consulting patients on digital platforms. For example, P22’s doctor had mistakenly prescribed him a lower dosage of medicine than what he needed during a Zoom consultation, which necessitated a visit to the clinic to obtain the correct dosage (Table 4). Participants were also appreciative if doctors were able to discern that their condition required a more detailed examination and that an in-person consultation was needed. For example, P24 expected doctors to know when to refer patients with severe medical symptoms to specialists for further medical assessment (Table 4). Moreover, participants would like doctors to perform a holistic examination of their conditions so as not to miss the symptoms of illnesses that could have otherwise been detected through physical examination. Participants such as P2 called for doctors to look out for signs that might reveal illnesses other than the one that the patient was seeking treatment for (Table 4). According to P2, doctors should know how to detect these through a thorough assessment of the patient’s body language and facial expression. In general, participants preferred teleconsultation for acute illnesses such as coughs and colds. Physical consultation was deemed necessary when seeking treatment for their chronic conditions.

When it comes to participants’ expectations of doctors’ communication skills when using EHRs, eye contact was appreciated but deemed nonessential by most participants (n=15, 60%). Generally, participants did not find it necessary for doctors to maintain regular eye contact with them, as they valued the doctors’ efficiency in carrying out their clinical work more than eye contact. To them, it was important for doctors to be competent and focused when documenting clinical records in order to avoid committing errors (Table 4). The older patients also shared that the rapport they already established with the doctor rendered sustained eye contact redundant (Table 4).

Although the participants did not regard regular eye contact during clinical documentation as essential, a significant proportion (n=21, 84%) opined that doctors should still display empathy in other phases of the clinical encounter regardless of whether DHTs were used. To participants such as P19, a patient with kidney disease, empathy should be conveyed even more in the digital age when DHTs were being used. P19 was concerned that doctors might not exhibit as much empathy if they were to use DHTs. This sentiment arose from her experience where even in the absence of DHTs, her doctor had not shown empathy toward her; the doctor had dismissed her pain and merely prescribed her medication after she shared about her skin condition (Table 4). A similar sentiment was shared by P25, a patient with paraplegia, who emphasized that empathetic communication, above any other skills, should be central to a doctor’s bedside manners. This expectation came about following her brief conversation with her doctor, who informed her that she could no longer walk. According to P25, the doctor did not even show any empathy or take the time to explain how he derived at his diagnosis (Table 4).

In addition, most participants from the age group of 20 to 39 years saw a greater immediate need for doctors to use digital technologies such as AI and machine learning for more accurate diagnosis and treatment than those in the older age groups who were having similar conditions. Participants from the former age group expected doctors to be trained in the algorithms behind these technologies, as well as the underlying principles and purposes of different DHTs in order to better assist them in diagnosis and treatment. For example, P15, a 28-year-old patient, who had encountered difficulties with venipuncture, shared that having a technological device would help doctors locate her veins and draw her blood without inflicting any pain on her. This method was preferred to the existing one where she had to be poked multiple times by her doctors based on trial and error. Similarly, P21, a 27-year-old patient, believed that AI technologies would help streamline certain aspects of the health care process, making diagnosis and treatment more time efficient. This expectation came about after her doctors took 1 year to diagnose her with rheumatoid arthritis. Previously, 4 doctors who attended to her had dismissed her condition, as the symptoms did not fit the regular markers of rheumatoid arthritis.

**Table 3:** Demographics of participants (N=25).

Characteristics		Participants, n (%)
**Gender**		
	Male	14 (56)
	Female	11 (44)
**Age (y), range**		
	20-39	8 (32)
	40-59	10 (40)
	≥60	7 (28)
**Ethnicity**		
	Chinese	12 (48)
	Indian	4 (16)
	Malay	8 (32)
	Other ethnicities	1 (4)
**Educational qualification**		
	Secondary	5 (20)
	Postsecondary: A level, diploma, ITE^a^, or other postsecondary qualification	8 (32)
	Bachelor’s degree	8 (32)
	Master’s degree	3 (12)
	PhD	1 (4)
**Housing type**		
	1-2 room HDB^b^	3 (12)
	3-room HDB	0 (0)
	4-room HDB	8 (32)
	5-room HDB	4 (16)
	HDB maisonette	2 (8)
	EC^c^	1 (4)
	Private condominium	4 (16)
	Landed property	3 (12)
**Primary medical conditions**		
	Cancer	4 (16)
	Cardiovascular diseases	3 (12)
	Diabetes	3 (12)
	Gout	3 (12)
	Kidney disease	3 (12)
	Neurological disease	3 (12)
	Osteoarthritis	3 (12)
	Rheumatoid arthritis	3 (12)
**Duration of primary medical condition (years)**		
	1-5	7 (28)
	6-10	8 (32)
	11-15	2 (8)
	16-20	2 (8)
	>20	6 (24)

^a^ITE: Institute of Technical Education.

^b^HDB: Housing & Development Board.

^c^EC: executive condominium.

**Table 4:** Illustrative quotes from interviews with participants.

Themes and subthemes		Quotes from participants
**Active and ethical engagement with medical data in mobile apps and wearable devices**		
	Guidance from and collaboration with doctors in the use of mobile apps	“I expect doctors to collaborate with patients and guide them on which app is safe to use since there are so many of them out there...One of the points I keep making at various conferences is that, patients and doctors are not really collaborators in Asia, we are more like in a donor-and-a-beneficiary relationship, where the doctor is giving something and we are just taking it. We are not rising up as a partner in care. We need to take some responsibility, you know, contribute, understand, and then work together.” [P5 aged 55 years, stroke survivor]
	Doctors’ knowledge of mobile apps	“I’ve used HealthHub app to check appointments and the results of my blood test, which will usually be out about two hours after the test. I will check my potassium level et cetera after taking blood at the hospital and before consultation. I expect the doctor to know how to use HealthHub too because it can help to speed up consultation with the doctor since both of us would have seen the health data already even before the consultation. There was once when I saw a young doctor for consultation and told her, ‘I already know my potassium level, et cetera from the blood test, and she was like, how did you know?’ So, I told her it’s all in my phone. She might not know it because the app was just launched back then.” [P15 aged 28 years, kidney and systemic lupus erythematosus]
	Doctors’ engagement with patients’ medical data found in mobile apps and wearable devices during consultation	“When using the device, I expect doctors to help me how to use these stuffs correctly, review the results and explain anomalies.” [P20 aged 39 years, diabetes, osteoarthritis, and rheumatoid arthritis]
	Doctors’ integrity when handling patients’ data	(On concern about data privacy): “My concern is with data security. Doctors should not send photos of scans to their colleagues on WhatsApp for a quick consult. It’s convenient but it’s not ideal because sometimes your WhatsApp account can be synced to your Google photos. So, everything just automatically gets backed up in their personal account, unless they go and delete it. If they don’t send the scan with our IC (Identification Card) number, it’s not so bad. So, it’s important to protect confidential information.” [P24 aged 39 years, thyroid cancer and rheumatoid arthritis]
	Doctors’ awareness of cybersecurity risks	(On concern about data privacy): “My concern is with data security. Doctors should not send photos of scans to their colleagues on WhatsApp for a quick consult. It’s convenient but it’s not ideal because sometimes your WhatsApp account can be synced to your Google photos. So, everything just automatically gets backed up in their personal account, unless they go and delete it. If they don’t send the scan with our IC [identification card] number, it’s not so bad. So, it’s important to protect confidential information.” [P24 aged 39 years, thyroid cancer and rheumatoid arthritis]
**Comprehensive and uncompromised quality of care**		
	Undivided attention	“I don’t think my expectations of the doctor would be the same when it comes to teleconsult. Yes, it’s kind of a replication of the clinical setting where we are talking to each other. But now, the doctor has to look at me and talk, right? Whereas when in the clinic, the doctor can be distracted by the computer, or saying something to the nurse or passing a note to whoever walks in. There are a lot of things happening in the clinical setting whereas in a zoom meeting, you are literally talking to that one person, so I’d expect there to be 100% attention.” [P1 aged 54 years, cancer]
	Pitfalls and limitations of teleconsultation	“In terms of preference, I still prefer face-to-face so that I can get appropriate care and treatment because when I do it over Zoom, the doctor cannot monitor my blood pressure and breathing. I’ve been given a lower dosage of medicine before when doing consultation over zoom. When that happened, I still needed to do a face-to-face consult anyway to take a higher dose of medicine.” [P22 aged 35 years, asthma and mitral valve prolapse]
	Knowledge of medical conditions that require further assessment	“I would expect the doctor to know when it’s time to escalate the situation to a specialist. So, if the symptoms are severe, they should refer the patient to urgent care.” [P24 aged 39 years, thyroid cancer and rheumatoid arthritis]
	Holistic assessment	“Before going into condition-specific assessment, I’d expect the doctor to check the patient’s overall health and mobility issues. Maybe for older patients, can get them to stand up, walk a few paces because based on their movements, doctors can detect a lot of other things. So, they shouldn’t just focus on their specialty, but examine the patients on other things as well, like their tone of voice, facial expression, and look out for signs that may tell their emotional issues, mental issues. So, doctors should have a protocol for these things before they go into the specifics.” [P2 aged 64 years, cancer]
**Efficiency outweighs eye contact for patients when doctors use EHRs^a^**		
	Doctors’ efficiency is valued by patients more than eye contact	“I don’t expect them to maintain eye contact the whole time because I understand that they need to see the computer, our medical conditions, and everything. It’s more important for them to know what’s happening to us. Maybe, eye contact is more important in the ward, but in the clinic, I don’t really mind if the doctor does not have eye contact with me.” [P13 aged 42 years, diabetes]
	Eye contact is appreciated but deemed nonessential	“Most doctors don’t have eye contact with the patient...when you know your doctor well and have confidence in the doctor, I don’t think it is necessary, though it would be good to have.” [P14 aged 75 years, gout]
**Empathetic communication regardless of DHT^b^ use**		
	Patients value human touch regardless of DHT use	“Even as doctors use digital technology, they still need to have that human touch. Based on my experience, I had a skin condition where there was a lump. When I went for consultation, the doctor did not even touch or see it. She just prescribed me antibiotics and asked me to come back for a one-week appointment. So, I think they should show more concern towards the patient, listen to our complaints and problems. Don’t just prescribe medicine and send the patient off. I was so upset. I don’t want other patients to go through the same experience because I was in pain and she didn’t even see to it. So, if without technology, they can already ignore the patient, what more if technology is present?” [P19 aged 56 years, kidney disease]
	Effective communication and bedside manners regardless of DHT use	“Even if doctors were to adapt digital technology in the health care setting, at the end of the day, it boils down to whether they can deliver a message or communicate a diagnosis to the patient empathetically or not...when relaying a message, doctors shouldn’t just approach the patient at the bed, and say, ‘Hey, I’d like to tell you that you are diagnosed with this condition,’ then just walk away without elaborating on the statement. It’s not helpful for the patient who is trying to process it mentally and who is not well not-versed in the condition. So, doctors should explain to the patient on how they derive at the diagnosis. When I was officially diagnosed with this disease, I couldn’t move my legs, my toes, I couldn’t move anything at all. The doctor just opened the curtain and told me, ‘I don’t think you can walk anymore,’ and just walked away. I was alone at that time, and just woke up from a one-and-a-half month coma, so I broke down there and then, non-stop. There was just so much emotions. It seems like when people go up the corporate ladder, they tend to neglect the humanistic aspect, the empathy.” [P25 aged 26 years, tuberculosis meningitis and pulmonary tuberculosis]
**Competence in using DHTs for clinical procedure and diagnosis**		
	Competence in using DHTs for venipuncture (younger participants)	“My veins are very fine, so I hope that in the future, there will be a machine or technology that can help locate my veins and poke them without having to poke a lot of times. Humans have to do it based on trial and error, and it’s very painful. Sometimes, my doctors have to poke me four or five times just to get the vein. So, if there is a machine that can poke once only and make sure there is blood in the veins, that would be cool. Otherwise, patients like me will suffer. Sometimes, when the senior doctors ask the junior doctors to try and poke me, they will poke multiple times. It is very painful!” [P15 aged 28 years, kidney and systemic lupus erythematosus]
	Competence in using DHTs to make accurate diagnosis (younger participants)	“Digital technologies are good; they can be predictive. Some doctors tend to stick to the books, so, they may not be able to find out the conditions as easily as they will when aided with digital technologies. When I told my doctors that I suspected I have rheumatoid arthritis, they said it’s not rheumatoid because my conditions don’t fit the definitive terms and criteria, like pain in the usual wear and tear areas. About four doctors including GPs and specialists did not think it was rheumatoid. But eventually, it was only when I had pain in my toes, on top of the usual markers, then the specialist diagnosed me with rheumatoid arthritis. So, using digital technologies to diagnose conditions will be helpful...machines are less likely to miss errors; they can pick them up better than doctors.” [P21 aged 27 years, rheumatoid arthritis]
	Accuracy and precision (younger participants)	“Digital technologies are good; they can be predictive. Some doctors tend to stick to the books, so, they may not be able to find out the conditions as easily as they will when aided with digital technologies. When I told my doctors that I suspected I have rheumatoid arthritis, they said it’s not rheumatoid because my conditions don’t fit the definitive terms and criteria, like pain in the usual wear and tear areas. About four doctors including GPs and specialists did not think it was rheumatoid. But eventually, it was only when I had pain in my toes, on top of the usual markers, then the specialist diagnosed me with rheumatoid arthritis. So, using digital technologies to diagnose conditions will be helpful...machines are less likely to miss errors; they can pick them up better than doctors.” [P21 aged 27 years, rheumatoid arthritis]
	Time efficiency (younger participants)	“Digital technologies are good; they can be predictive. Some doctors tend to stick to the books, so, they may not be able to find out the conditions as easily as they will when aided with digital technologies. When I told my doctors that I suspected I have rheumatoid arthritis, they said it’s not rheumatoid because my conditions don’t fit the definitive terms and criteria, like pain in the usual wear and tear areas. About four doctors including GPs and specialists did not think it was rheumatoid. But eventually, it was only when I had pain in my toes, on top of the usual markers, then the specialist diagnosed me with rheumatoid arthritis. So, using digital technologies to diagnose conditions will be helpful...machines are less likely to miss errors; they can pick them up better than doctors.” [P21 aged 27 years, rheumatoid arthritis]
	Concern on the reliability of technology (older participants)	“Retirees like me pick up things much slower than the younger generation, who use technology more regularly. I am open to technology, but the thing about technology is that if the technology goes bust, then all the data in the device will be gone. All my appointments will be lost. If you were to lose electricity, or the technology breaks down, then you may not get back the data. I am also open to robots diagnose my conditions or treat me, but I am concerned about what the robots will come up with. So, we shouldn’t be too reliant on technology...when it comes to diagnosis, I’ll be quite skeptical of course if a robot were to diagnose me. Eventually, you still need a doctor to oversee the diagnosis the technology has come up with.” [P14 aged 75 years, gout]
	Concern of trust on the reliability of diagnosis if it is based on DHTs alone (older participants)	“I’m not open to technology because technologies like AI do not have emotions like humans do. Even if it helps to improve the accuracy of diagnosis, I’m still not open to it because what if there is no electricity? You can’t just depend on technology. It can trip anytime. If there’s a trip, everything will be gone and doctors won’t know what to do.” [P19 aged 56 years, kidney disease]

^a^EHR: electronic health record.

^b^DHT: digital health technology.

Compared to the younger participants, those from the older generation saw less need for the use of DHTs. They were concerned about the reliability of diagnosis, the perceived absence of human touch, and the risk of losing their medical data in the event of a technological breakdown if they were to be treated with DHTs. This was expressed by participants such as P14, a 75-year-old patient, and P19, a 56-year-old patient (Table 4). P14 and P19 continued to favor the diagnosis made by doctors over that made by DHTs due to their greater trust in the expertise of medical professionals. They would also opt for diagnosis and treatment advised by doctors over what is suggested DHTs, as the former was understood to offer emotional connection and understanding, which were lacking in the latter. Moreover, older participants were generally skeptical about the reliability of technology. Relatedly, they had concerns about the doctors’ ability to recover their medical data should there be a system failure or technical malfunction.

Overall, the findings indicated that age, compared to other social determinants, was more influential in differentiating the participants’ health care experiences and expectations, at least in this study. By gathering the views of the patients who were seeking medical care from outpatient clinics and who had sought treatment in the wards, this study sheds light on the importance of considering their health care experience and expectations to ensure that their needs were not being ignored. Interviewing the patients who had various medical conditions also highlights the type of digital technology that would be useful for specific health care purposes.

## Discussion

### Principal Findings

Overall, the participants expected doctors to be competent in using different DHTs. With regard to apps and wearables, they would like doctors to integrate data from these devices into doctor-patient communication and to handle their data ethically. When it comes to telemedicine, they expected doctors to deliver comprehensive and high-quality care that does not compromise their safety. According to them, doctors should be knowledgeable enough to identify the type of medical conditions that were appropriate for online consultation and those that needed further assessment. In addition, while participants did not perceive regular eye contact when using EHRs as essential, they still valued the doctor’s display of empathy in other phases of the clinical encounter, regardless of DHT use. The younger participants also expected doctors to be trained in the algorithms, principles, and purposes of DHTs such as AI to better assist them in the diagnosis and treatment processes. By interviewing patients, we have obtained the perspectives of an important yet often overlooked stakeholder in the health care system.

To our knowledge, this is the first study to assess patient perspectives of the existing gaps in doctors’ clinical competencies when deploying DHTs. Our findings are unique, as the medical conditions among the study participants were varied. Previous studies have either explored DHT deployment among adults with specific single chronic conditions, such as hypertension, diabetes, and chronic heart failure or are not focused on patients’ expectations of doctors’ clinical skills [12,40-42]. Interviewing participants with various chronic conditions has offered us insights into their preferred mode of consultation; while teleconsultation was preferred for acute illnesses, physical consultation was still deemed necessary for chronic conditions. It also proved that the need to equip doctors with DHT competencies for better diagnosis and treatment was not specialty dependent but was something that needed to be implemented across the health care sector.

One similarity between the findings of this study and those of others is that DHTs are not the main driver of health-seeking behavior among older adults [12,40]. In general, they prefer receiving traditional health care services from medical professionals to exclusively relying on DHTs. However, unlike other studies indicating that older adults with lower educational qualifications (primary or secondary level) are less receptive and less likely to use DHTs [12,40], our research suggests that participants’ limited receptiveness is not necessarily correlated with their educational qualifications. Those with postsecondary education and above also expressed reluctance to use DHTs. Our results therefore offer a nuanced perspective on patients’ attitudes toward technology.

By conducting qualitative interviews with patients, this study has uncovered diverse views and informed future studies about the need to avoid associating perceived attitudes with specific social identities at the outset of research. This may only run the risk of perpetuating stereotypes about people belonging to certain identities. As the findings have shown, patients’ expectations and concerns need not be differentiated by their race, gender, or socioeconomic status. Rather, factors such as age and type of medical conditions tend to be more salient, proving the value of our constructivist and inductive approaches.

Unlike past qualitative studies, the lack of sustained eye contact between the doctor and patient during clinical documentation did not seem to matter to the participants of this study [43-46]. This could be attributed to the transactional relationship between the doctor and patient that characterizes many clinical encounters in Singapore. This type of care recognizes that the patient has a specific need, diagnoses and treats the condition, controls the risk factor, and makes a referral [47]. The quality of care tends to be determined by the ability of the doctor to abide by a set of prescribed guidelines that makes him or her a “good” doctor [47]. This differs from relationship-based care, which tends to focus on the quality of interaction between the doctor and patient [47]. The omnipresence and relative accessibility of doctors in Singapore compared to those in other countries may explain this taken-for-granted aspect of relationship [48]. Furthermore, in a fast-paced country like Singapore where efficiency and accuracy in the management of medical conditions are highly valued [48,49], the lack of eye contact seems like a characteristic that patients are willing to forego. Nonetheless, these should not compromise the humanistic and empathetic practice of medicine. As our participants such as P2 have expressed, appropriate clinical inquiry is still deemed important when DHTs are used. This is reiterated by the existing studies, which prove that attentiveness to the computer screen rather than eye contact with the patient during clinical encounters does adversely affect doctors’ psychosocial inquiry, emotional patient responsiveness, and full patient disclosure [46,48].

Some of the findings of this study have also been reported by other countries. These include the importance of identifying the cases that are appropriate for teleconsultation. Past reviews and studies have also reported the need for doctors to be equipped with the knowledge of selecting patients for teleconsultation so as not to compromise their safety [50-52]. However, many of these studies are based on individual medical conditions that are deemed stable. Future research should also consider the selection and identification procedures of patients with comorbidities and complex conditions since these are becoming increasingly common worldwide [53].

The value of incorporating training in AI technologies early in medical education has also been recognized in a large body of works. Examples of AI competencies include knowing the limitations, risks, and medicolegal aspects of AI [54-56]. In addition, concerns about privacy and data intrusion when DHTs are used are also commonly reported by other studies [7,12]. To illustrate, a study conducted with residents, patient representatives, and health care providers at a health facility catchment in Sydney, Australia, reported that both groups expressed concerns with safety issues such as data safety and privacy and the risk of hacking when telemedicine is used [7].

Other works have also recognized the value of integrating data from DHTs into the interaction with patients [6,57]. For example, the study by Loos and Davidson [57] that assessed doctors’ views on the potential integration of wearables into patient care showed how effective doctor-patient communication aided by wearables serves a few purposes. These include forging strong interpersonal relationships between the doctor and patient and getting accurate information from patients to inform diagnosis, treatment decisions, and overall management of health [57]. However, as shown by the scoping review conducted by Hilty et al [58], there is currently a lag in the clinical, technological, and administrative workflow at the international level with regard to incorporating sensors, wearables, and remote patient monitoring in clinical care [58]. Hence, standardized frameworks of competencies need to be developed for doctors to effectively engage patients with these devices.

Moreover, our findings reiterate those of other studies when it comes to how age affects the use of DHTs. Specifically, participants who belong to the advanced age bands did not perceive an urgent need for their doctors to deploy DHTs for their medical conditions, unlike the younger ones. This is exemplified by the qualitative study by Low et al [12] of how older adults in Singapore negotiate DHTs in their everyday lives. In this study, the authors discovered not only a low uptake of DHTs among those aged 50 to 65 years but also the lack of perceived immediate need for them to use these technologies despite their willingness to adopt them. This is mainly attributed to the lack of technology-centeredness in their health-seeking behavior [12]. Another study corroborates this by demonstrating how adults from *Generation X*, defined as those born between 1943 and 1960, required technology training more than the *millennial generation*, referring to those born between 1982 and 2000. This is in view of the Generation X’s lower comfort level with using DHTs, highlighting their lesser dependence on and reluctance to use DHTs [1]. Hence, initiatives to increase the awareness and acceptance of DHTs among the population are needed if they were to be implemented nationwide.

### Recommendations

The findings have reiterated the importance of exploring the views of patients so that their needs and the competencies of doctors can be more aligned in this digital age. This section offers recommendations on how each of the participants’ expectations and concerns can be addressed accordingly.

### Engaging Data in Apps and Wearables Effectively With Patients

To fulfill patients’ expectations of having their doctors communicate the meaning of their medical data with them, protected time is needed for doctors during consultations. On the basis of the participants’ sharing, time constraints and a hectic work environment seem to be major barriers that prevent doctors from communicating effectively with patients. Hence, having protected time would allow them sufficient time to offer the necessary advice to their patients on the use of DHTs and provide adequate explanations of the data. Health care institutions can work with designers of technology to put in place a system that works like a voice-to-text tool where communication between the doctor and patient can be captured and transcribed in real time. This would help reduce doctors’ time on clinical documentation, allowing them more time to interact with patients and respond appropriately to any of their concerns.

In view of the evolving model of care where the traditional model of the doctor-patient relationship is being replaced by shared decision-making between the doctor and patient, doctors should also act as expert partners in patients’ health care journey. In this regard, it is important to consider the study by Mesko et al [59], which highlights that driving behavioral change for patients entails not only just technological shifts but also a consideration of humanistic elements. Specifically, more opportunities need to be created to educate patients about DHTs [59]. For example, doctors can advise their patients on how they can use DHTs in a safe and effective manner during consultations. These include identifying and downloading legitimate apps and teaching them how to access their medical data. This would not only expand patients’ knowledge of DHTs but also enhance the doctor’s commitment to supporting their overall well-being.

### Determining Suitable Medical Conditions for Teleconsultations

To train doctors in holistic assessment of patients’ conditions during teleconsultation, as well as in determining conditions suitable for this type of consultation, a comprehensive set of guidelines needs to be developed at the international level. Reviews on telemedicine have shown that there is a lack of guidelines and standards for implementing telemedicine both at the international and national levels [60,61]. Although many telemedicine reports have highlighted the need to develop these guidelines, only a few exist in practice [60]. Current telemedicine guidelines for clinical practice worldwide have been limited to specific specialties such as psychiatry, dermatology, pathology, and radiology [60]. A policy review on telemedicine in the Southeast Asia region of the World Health Organization also reveals significant variations in the adoption and implementation of telemedicine guidelines among 11 Southeast Asia region countries of the World Health Organization [57,61]. Among the 11 countries, only 5 have developed telemedicine guidelines including India, Bangladesh, Thailand, Indonesia, and Nepal [61].

In Singapore, the country’s Medical Council and professional bodies have developed codes and guidelines to regulate the use of telemedicine [62,63]. However, the considerations for delivering care via telemedicine are outlined only broadly. Specifically, the reasonableness of conducting teleconsultations is determined by “the clinical context, the clinical objectives and the compatibility of technology to meet those objectives” [62]. The delivery of care using telemedicine is based on general Clinical Practice Guidelines [62]. In addition, there are no specific guidelines to determine the type of cases that are eligible for telemedicine consultation. Currently, the diagnosis, prescription of medicine, and issuance of medical certificates via telemedicine depend on the “professional judgement of the relevant doctor” and the “specific facts and circumstances of each presenting case” [63].

To ensure the standardization of current work practices and guidelines, some authors have highlighted the important role of international telemedicine organizations [60]. For example, organizations such as the American Telemedicine Association and the United Kingdom’s Telemedicine and eHealth Forum of the Royal Society of Medicine can take the lead by defining telemedicine guidelines under the direction of clinicians with relevant telemedicine expertise [60]. These clinicians should come from different medical specialties so that the health care needs of patients with comorbidities can be fulfilled.

### Practicing Empathetic Communication When Using DHTs

To ensure that empathetic communication is not compromised when doctors are using DHTs, they can be trained to practice this skill alongside the adoption of DHTs. For example, during virtual consultations, doctors can practice active listening when communicating with patients. This involves giving patients their full attention and conveying attentiveness through nonverbal cues such as facial expressions and body language. These skills can help build a connection with patients.

A systematic review on how compassion is discussed in relation to AI technologies in health care shows that there are different ways in which AI can promote compassion. These include enhancement of the empathetic awareness of patients’ suffering, empathetic response and relational behavior, communication skills, health coaching, therapeutic interventions, moral development learning, clinical knowledge and assessment, health care quality assessment, therapeutic bond and alliance, and provision of health information and advice [64]. However, most of these studies discuss how AI technologies can be used to train health care professionals and trainees to deliver clinical care using virtual platforms and patients. Further research on how DHTs such as EHRs, apps, and wearables can be leveraged during clinical consultations with real patients needs to be conducted.

At present, the framework proposed by Loos and Davidson [57] of the competencies for clinicians and trainees when using sensors, wearables, and remote monitoring devices may serve as a guide in determining the necessary competencies. This framework is organized according to 3 learner levels, namely, novice or advanced beginner, competent or proficient, and advanced or expert levels and is based on the domains outlined by the Accreditation Council of Graduate Medical Education (ACGME) [57]. Beyond ways to embody interpersonal and good communication skills, it also outlines how clinicians and trainees can deliver quality care through their knowledge of these DHTs, for example, learning the types of diseases that can be treated with wearables (ACGME milestone levels 1-2); developing a plan to review, communicate, and deliberate on data (ACGME milestone levels 3-4); and researching new ways to improve care using DHTs such as AI (ACGME milestone level 5). Standardized evaluation measures may help to determine the effectiveness of such frameworks.

### Using AI Technologies Competently and Ethically

To train doctors to be competent in DHTs such as AI, professional bodies can offer courses and training programs to doctors through continuing medical education, as has been done by the Digital Medicine Society in partnership with Rocky Vista College of Medicine and the American College of Osteopathic Family Medicine in the United States [65]. Such programs can help educate and encourage health care professionals to embrace digital medicine and train them in the benefits, risks, and pitfalls of AI. Another way to encourage the adoption of DHTs among health care professionals is to share published data on studies that involve large patient cohorts and that report on the accuracy of well-documented DHTs.

To address the concerns about possible intrusions of personal data, a regulatory framework that understands the workings of technological innovations and their potential lapses needs to be introduced in order to prevent the leakage of sensitive information. In addition, tighter laws on data breaches need to be legislated to improve public trust. In the United States, laws such as Genetic Non-Discrimination Act serve to defend patients from unauthorized third-party access to data [59]. More such laws need to be devised and implemented to increase public trust in DHTs. Doctors and medical trainees also need to be trained in handling medical data ethically in order to safeguard patients’ confidential information. The proliferation of medical data breaches in recent years caused by cyberattacks and ransomware attacks in Singapore and around the world necessitates tighter laws to protect patients’ medical data.

### Strengths and Limitations

This qualitative study informs us about the expectations and concerns of patients with DHTs. By interviewing participants from different social and economic backgrounds, as well as medical conditions, the sample achieved diversity in narratives, and the study benefited from the rich data. Seeking the opinions of patients from outpatient clinics, most of whom had been hospitalized for the same conditions, also allows us to uncover their experiences in both the inpatient and outpatient settings. In addition, conducting qualitative interviews enables us to gain in-depth insights into patients’ experiences and expectations and place their narratives at the forefront of our research.

A perceived limitation of this study is its small sample size. This may limit the generalizability, validity, and reliability of the study. With a small sample size, it is challenging to generalize the findings to a larger population. The findings may only be applicable to the individuals included in the study and may not represent broader groups [21]. Furthermore, a small sample size may not adequately capture the diversity within the population of interest [21]. Consequently, the findings may lack depth and breadth, leading to a limited understanding of the phenomenon under investigation. In addition, small sample sizes increase the risk of selection bias in the recruitment strategy. Researchers may inadvertently select participants who are more accessible or willing to participate, thus leading to a biased sample that does not accurately represent the population.

Nonetheless, as with other qualitative studies that adopt interviewing techniques, an in-depth analysis of participants’ narratives offers a contextualized understanding of their expectations and concerns. Furthermore, although not entirely generalizable, the findings from this study would bear important implications for digital health training programs, initiatives, and frameworks in other developed countries. As a scoping review on digital health competency frameworks for health care professionals has shown, frameworks for training doctors and medical trainees in relevant digital competencies are still lacking [66].

Another limitation lies in the lack of language diversity among participants, all of whom happened to be English-speaking. Their English language proficiency is likely attributed to the widespread use of English as the official language of communication in Singapore. Having research participants come from a homogenous linguistic background may restrict the ability to apply the findings to diverse communities where other languages are spoken. Since language is intricately linked to culture and educational background [8], this may also exclude the influence of cultural and class factors on patient expectations. Despite the language limitation, participants came from diverse ethnic, educational, and socioeconomic backgrounds.

In addition, we acknowledge that there are other digital technologies that are not discussed in this study, given its focus on the type of technologies that have been introduced for clinical practice in Singapore. Therefore, future research should also examine how more recent AI technologies such as ChatGPT could potentially transform health care from the perspectives of different stakeholders including patients and health care professionals. ChatGPT is an AI-powered conversational agent developed by OpenAI. It is based on the GPT architecture. Designed to understand and generate humanlike text based on the input it receives, it has the potential to revolutionize health care delivery and services in several ways. These include promoting healthier lifestyle habits through personalized health coaching and interventions and serving as a clinical decision support tool for health care providers. The latter may include offering real-time access to evidence-based guidelines, medical literature, and treatment protocols. Such technologies raise the question of how medical diagnosis, treatment plans, and patient management may evolve in the future.

### Conclusions

This study has explored the clinical encounters of patients with chronic illnesses amid the increasing digitalization of health care. Evaluating their perspectives proves crucial, as they can be considered a key facilitator for technology implementation and enhancement in health care settings. By identifying their expectations of doctors’ clinical competencies, this study has shown that traditional and basic clinical skills such as effective communication, clinical reasoning, history taking, physical examination, and procedural skills should neither be neglected nor compromised in the digital age. Nonetheless, the role of clinicians needs to evolve with the introduction of DHTs. For example, the way in which DHTs are transforming the traditionally paternalistic rapport between the doctor and patient needs to be considered. With patients now holding the power to assess the standard of health care received and being more involved in the decision-making processes of their own health [59], an expansion of doctors’ clinical competencies is needed in order to consider these shifts.

In addition, the findings of this study might inform the health care practices and policies in other high-income countries. These include prioritizing the integration of digital health care education into medical training programs to equip doctors with the necessary competencies. Examples of initiatives may include updating the curricula of medical schools, offering continuing professional development opportunities, and fostering a culture of lifelong learning among health care professionals. In addition, governments and health care systems should allocate resources toward implementing DHTs that align with patient expectations and preferences. They could also implement patient engagement and education initiatives to increase the awareness and acceptance of DHTs among the population. Moreover, policy makers should develop regulations and guidelines to govern the use of DHTs in health care delivery including ensuring data privacy and security and addressing issues related to liability. Overall, the findings from this research could serve as a road map for other high-income countries to leverage the potential of DHTs in enhancing health care delivery in the digital age.

## Abbreviations

ACGME: Accreditation Council of Graduate Medical Education
AI: artificial intelligence
DHT: digital health care technology
EHR: electronic health record
